# Selective use of adjuvant chemotherapy for rectal cancer patients with ypN0

**DOI:** 10.1007/s00384-014-1831-0

**Published:** 2014-01-29

**Authors:** Kai-yun You, Rong Huang, Pei-rong Ding, Bo Qiu, Guan-qun Zhou, Hui Chang, Wei-wei Xiao, Zhi-fan Zeng, Zhi-zhong Pan, Yuan-hong Gao

**Affiliations:** 1 State Key Laboratory of Oncology in South China, Sun Yat-sen University Cancer Center, Guangzhou, Guangdong 510060 China; 2 Department of Radiation Oncology, State Key Laboratory of Oncology in South China, Sun Yat-sen University Cancer Center, Guangzhou, Guangdong 510060 China

**Keywords:** Rectal neoplasms, Chemoradiation, ypN0, Adjuvant chemotherapy

## Abstract

**Background:**

The administration of adjuvant chemotherapy for rectal cancer patients with ypN0 is controversial. The purposes of this study were to evaluate the role of adjuvant chemotherapy in ypN0 patients and to optimize its use for these patients.

**Methods:**

We performed a retrospective study of 160 rectal cancer patients who had the final pathology of ypN0 between March 2003 and November 2010. Overall survival (OS), disease-free survival (DFS), local recurrence-free survival (LRFS), and distant metastasis-free survival (DMFS) were compared between patients who did and did not receive adjuvant chemotherapy. Multivariate analysis was performed to explore clinical factors significantly associated with DFS, LRFS, and DMFS.

**Results:**

For ypT0–2N0 patients, the 5-year OS, DFS, LRFS, and DMFS were similar between patients who did and did not receive adjuvant chemotherapy (*P* > 0.05). For patients with ypT3–4N0, those who were given adjuvant chemotherapy exhibited a higher 5-year OS than those who were not (*P* = 0.026), with also an extended 5-year DFS (*P* = 0.050). Further analysis indicated that adjuvant chemotherapy could decrease the rates of distant metastases for ypT3–4N0 patients with no impact on local control. In multivariable analysis, both the final pathological stage and adjuvant chemotherapy were independent predictors of DMFS for the whole group. When stratified by pathological stage, adjuvant chemotherapy was still significantly associated with DMFS in the ypT3–4 stratum.

**Conclusions:**

Adjuvant chemotherapy may not improve survival for ypT0–2N0 patients. However, it may be clinically meaningful for ypT3–4N0 patients by decreasing rates of distant metastases. Further randomized controlled clinical trials are needed to address this problem.

## Introduction

Neoadjuvant chemoradiation (CRT) followed by radical resection is now recommended for locally advanced rectal cancer [1], which brings meaningful tumor shrinking, down staging, improved local control, and increased probability of sphincter-sparing surgery [2–5]. However, the degree of response to CRT, with its clinical significance in predicting the long-term outcome, varied among patients [6]. Patients who exhibited favorable response (ypT0–2N0) were reported to achieve excellent survival regardless of receiving adjuvant chemotherapy or not. Postoperative adjuvant chemotherapy, though typically recommended for all by NCCN guidelines, seemed to contribute little to improve the survival for these patients [6–10].

Currently, the delivery of adjuvant chemotherapy for rectal cancer patients undergoing chemoradiation is not evidence based [11]. Several studies have questioned the need of adjuvant chemotherapy for ypN0 patients, especially for those with ypT0–2 [8–10, 12]. However, it is still a controversial problem [8–10, 12–14]. The purpose of this study was to re-evaluate the role of adjuvant chemotherapy in patients with ypN0. We explored some clinical factors in predicting the prognosis for patients in this subgroup.

## Materials and methods

### Ethics statement

This research was approved by the Ethics Committee of Sun Yat-sen University Cancer Center, and the written informed consent was obtained from every patient included in the study.

### Patients

We conducted a retrospective study of 237 patients who had undergone CRT followed by surgery at the cancer center of Sun Yat-sen University between March 2003 and November 2010. All the patients had locally advanced rectal cancer as shown by biopsy. There were 27 patients excluded for concurrent distant metastasis, concurrent malignancy, or prior history of radiotherapy to the pelvis. Finally, 160 patients with pathology of ypN0 were enrolled in this study.

### Evaluation

Before CRT, all patients underwent staging workup, which included endorectal ultrasound, computed tomography (CT), magnetic resonance imaging (MRI), and chest radiography. Endorectal ultrasound was typically recommended for patients for its accurate T staging in our cancer center. Abdominal CT and chest radiography are routine examinations with at least CT or MRI for the pelvis. Pre-CRT Serum CEA, complete blood count, and liver function test were also measured.

### Treatment

Patients underwent a standard protocol of CRT with two courses of concurrent chemotherapy. The prescription dose to the whole pelvis was 46 Gy in 23 fractions over 5 weeks. The technique of radiotherapy was based on a three-dimensional conformal radiotherapy treatment planning system (PINNACLE 8) with a three-field irradiation plan being used (8-MV photon posterior–anterior field and 15-MV photon opposed lateral beams). The clinical target volume (CTV) included primary rectal tumor, peri-rectal tissues, presacral lymph nodes, internal iliac lymph nodes, and obturator lymph nodes. The superior border of the CTV was the bottom of L5, and the inferior border was 2.5–3 cm distal to the tumor. The anterior border was the posterior margin of the bladder or uterus, and the posterior border was the anterior margin of the sacrum. PTV is defined as CTV + 8∼10 mm.

The regimens of concurrent chemotherapy were Folfox6 and Xelox. There were 37 patients who were treated with chemotherapy of Folfox6 (oxaliplatin 85 mg/m^2^ day 1 + leucovorin 400 mg/m^2^ day 1 + 5-FU 400 mg/m^2^ iv day 1, then 2,400 mg/m^2^ civ 46–48 h), while the other 123 patients received Xelox (oxaliplatin 100 mg/m^2^ day 1 + capecitabine 1,000 mg/m^2^ bid, po, days 1–14).

At a median of 43 days (range, 20–73) after the completion of chemoradiotherapy, radical surgery for rectal cancer was implemented. All the operations were performed according to the TME-principles by colorectal surgeons, and the methods included low anterior resection, abdominoperineal resection, and Hartmann. Negative circumferential resection margin proved by pathology was achieved by all the patients in this study.

Adjuvant chemotherapy were administrated in 115 patients with the regimens of Folfox6 (oxaliplatin 85 mg/m^2^ day 1 + leucovorin 400 mg/m^2^ day 1 + 5-FU 400 mg/m^2^ iv day 1, then 2,400 mg/m^2^ civ 46–48 h), Xelox (oxaliplatin 130 mg/m^2^ day 1 + capecitabine 1,000 mg/m^2^ bid, po, days 1–14), and single agent capecitabine (capecitabine 1,250 mg/m^2^ bid, po, days 1–14). The median month of adjuvant chemotherapy was 4 (range, 2–7.5). The other 45 patients were not scheduled with adjuvant chemotherapy due to various reasons such as, severe postoperative complications, poor performance status, or refusal due to elder age.

### Pathological classification

Pathological tumor staging of the resected specimen was performed by experienced pathologists. The operative specimens of 160 patients were restaged by two independent pathologists according to the American Joint Committee on Cancer (AJCC) 7th edition staging system. If their stagings were inconsistent, then a third pathologist was needed to perform the task. All the specimens were carefully dissected by the pathologists to achieve all the potential lymph nodes, and the median of retrieved lymph nodes was 8 (range, 3–37).

### Toxicity assessment for neoadjuvant chemoradiation and adjuvant chemotherapy

Therapy-related adverse events were defined as complications that occurred during treatment, which were graded by using the Cancer Institute Common Terminology Criteria for Adverse Events version 3.0. Severe adverse events were defined as any grade ≥3 toxicity. Adverse events were recorded for each patient treated in our cancer center and were documented in our colonrectal database.

### Follow-up

The follow-up policy was every 3 months for the first 2 years after surgery and every 6 months thereafter. However, during the period of postoperative adjuvant chemotherapy, the follow-up was delayed until the completion of all treatments. Evaluations included complete blood count, liver function test, CEA, CA19-9, and physical examination during each visit. Chest radiography, CT scanning of the abdomen and pelvis, and colonoscopy were conducted every 6 months. Every follow-up for each patient was recorded in our database. In this study, the median follow-up period for all patients was 46 months (range, 18–101).

### Statistical analysis

All statistical analyses were performed by SPSS software, version 17.0. Categorical variables were analyzed by using the chi-square test or Fisher’s exact test. Continuous variables were analyzed by the Student *t* test or Mann–Whitney *U* test. The Kaplan–Meier method was employed to compare DFS rates and OS rates. Multivariate analysis of DFS, LRFS, and DMFS was performed by Cox proportional hazards regression, and the Cox proportional hazards model was performed using a forward conditional selection of variables. Variables with *P* value < 0.2 were entered into a Cox model. *P* < 0.05 was considered to be statistically significant.

## Results

### Clinical characteristics

There were 160 patients who completed CRT and were node-negative on the final pathology enrolled in our study. Among them, 115 patents received postoperative adjuvant chemotherapy, while 45 patents did not. Compared to patients who received adjuvant chemotherapy, those who did not were significantly older (*P* < 0.001) and more likely to receive the concurrent chemotherapy of Folfox6 (*P* = 0.011). Other variables such as gender, Hb, CEA, location of tumor, clinical tumor stage, clinical T stage, clinical N stage, tumor grade, interval between completion of chemoradiotherapy and surgery, type of surgery, number of retrieved lymph nodes, ypT stage, and follow-up were similar between two groups (Table 1).

**Table 1:** Patient demographics, baseline tumor characteristics, type of surgery, and pathological outcome

Variable	Adjuvant-chemo group	No adjuvant-chemo group	*P* value
Age, year	–	–	<0.001
Median	54 (15–80)	62 (39–77)	–
Gender	–	–	0.552
Male	87	32	–
Female	28	13	–
Hb, g/L	–	–	0.575
Average	129 (78–170)	126 (76–160)	–
CEA, μg/mL	–	–	0.081
Median	4.95 (0.20–157.50)	3.33 (0.54–249.60)	–
Location of tumor, from AV	–	–	1.000
≥7.0 cm	20 (17.4 %)	7 (15.6 %)	–
<7.0 cm	95 (82.6 %)	38 (84.4 %)	–
Clinical T stage	–	–	1.000
T1	2 (1.7 %)	0 (0.0 %)	–
T2	3 (2.6 %)	1 (2.2 %)	–
T3	46 (40.0 %)	18 (40.0 %)	–
T4 (T4a and T4b)	64 (55.7 %)	26 (57.8 %)	–
Clinical N stage	–	–	0.612
N0	41 (35.7 %)	20 (44.4 %)	–
N1	30 (26.1 %)	10 (22.2 %)	–
N2	44 (38.2 %)	15 (23.4 %)	–
Clinical stage	–	–	0.366
II	41 (35.7 %)	20 (44.4 %)	–
III	74 (64.3 %)	25 (55.6 %)	–
Tumor grade	–	–	0.743
G1	9 (7.8 %)	4 (8.9 %)	–
G2	87 (75.7 %)	36 (80.0 %)	–
G3	19 (16.5 %)	5 (11.1 %)	–
Concurrent chemotherapy	–	–	0.011
Folfox6	20 (17.4 %)	17 (37.8 %)	–
Xelox	95 (82.6 %)	28 (62.2 %)	–
Interval, day	–	–	0.588
Median	42	44	–
Type of surgery	–	–	0.734
Mile’s	56 (48.7 %)	20 (48.9 %)	–
Dixon	58 (50.4 %)	22 (48.9 %)	–
Hartmann	1 (0.9 %)	1 (0.2 %)	–
Retrieved lymph nodes			
Median	8	7	0.234
ypT stage	–	–	0.603
ypT0	39 (33.9 %)	13 (28.9 %)	–
ypT1	2 (1.7 %)	3 (6.7 %)	–
ypT2	24 (20.9 %)	10 (22.2 %)	–
ypT3	37 (34.8 %)	16 (35.6 %)	–
ypT4a	10 (8.7 %)	3 (6.7 %)	–
Follow-up, months	–	–	0.710
Median	47	41	–

Tumor grade: G1 = well differentiated, G2 = moderately differentiated, G3 = poorly differentiated

*Adjuvant-chemo* adjuvant chemotherapy, *Hb* hemoglobin, *CEA* carcinoembryonic antigen, *AV* anal verge

### Survival analysis

**Fig. 1:**
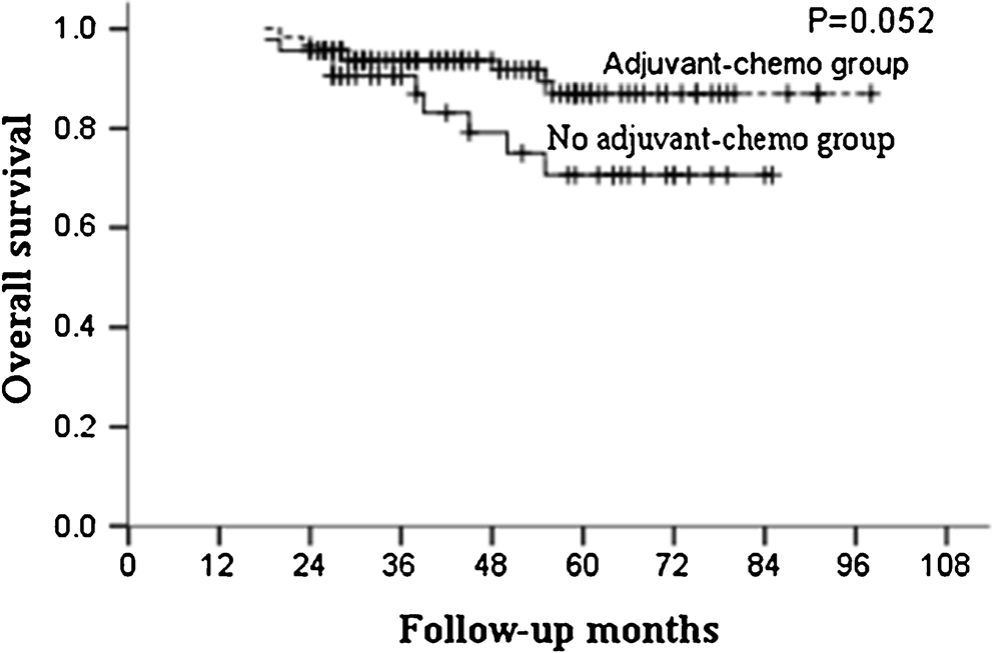
OS for the ypT0–4N0 patients stratified by treatment with adjuvant chemotherapy. No significant difference was found in OS between adjuvant-chemotherapy and no adjuvant-chemotherapy groups for ypT0–4N0 patients (*P* = 0.052)

**Fig. 2:**
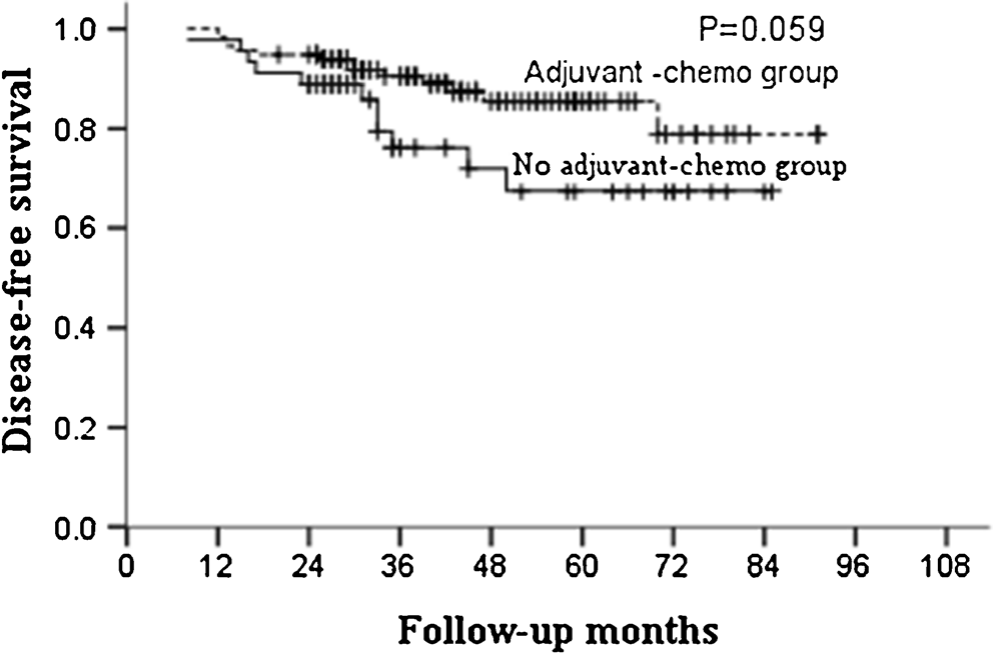
DFS for the ypT0–4N0 patients stratified by treatment with adjuvant chemotherapy. No significant difference was found in DFS between adjuvant-chemotherapy and no adjuvant-chemotherapy groups for ypT0–4N0 patients (*P* = 0.059)

**Fig. 3:**
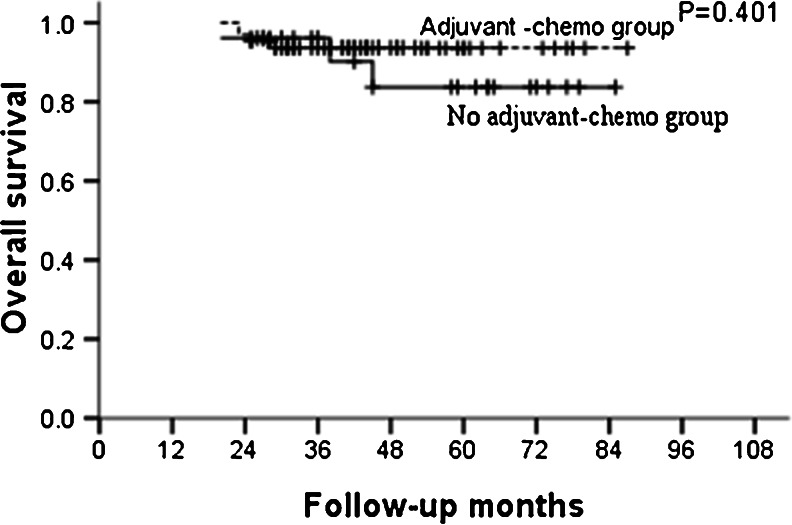
OS for the ypT0–2N0 patients stratified by treatment with adjuvant chemotherapy. No significant difference was found in OS between adjuvant-chemotherapy and no adjuvant-chemotherapy groups for ypT0–2N0 patients (*P* = 0.401)

**Fig. 4:**
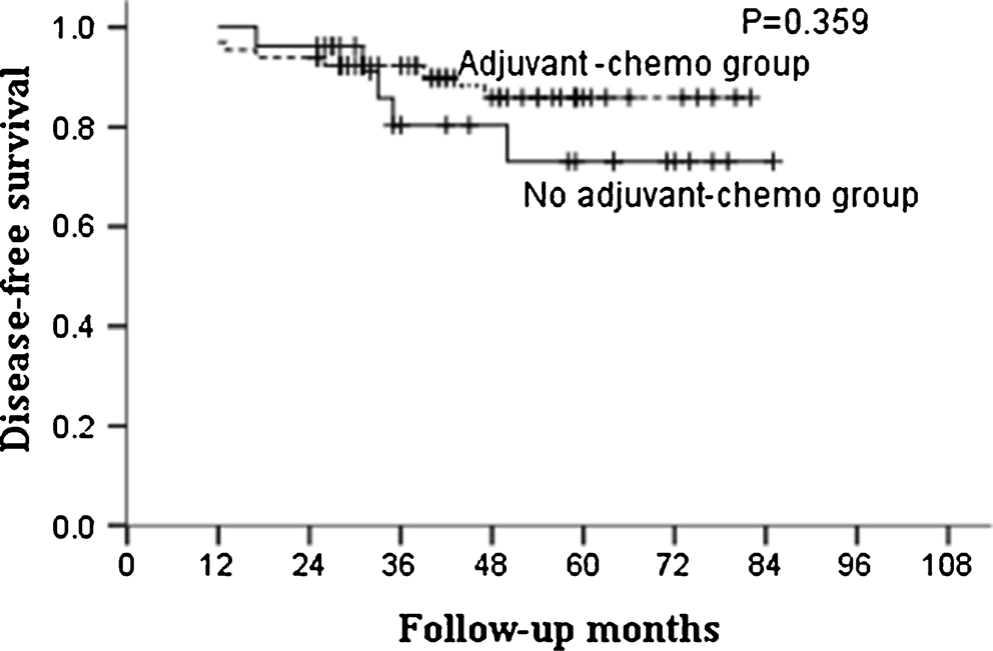
DFS for the ypT0–2N0 patients stratified by treatment with adjuvant chemotherapy. No significant difference was found in DFS between adjuvant-chemotherapy and no adjuvant-chemotherapy groups for ypT0–2N0 patients (*P* = 0.359)

**Fig. 5:**
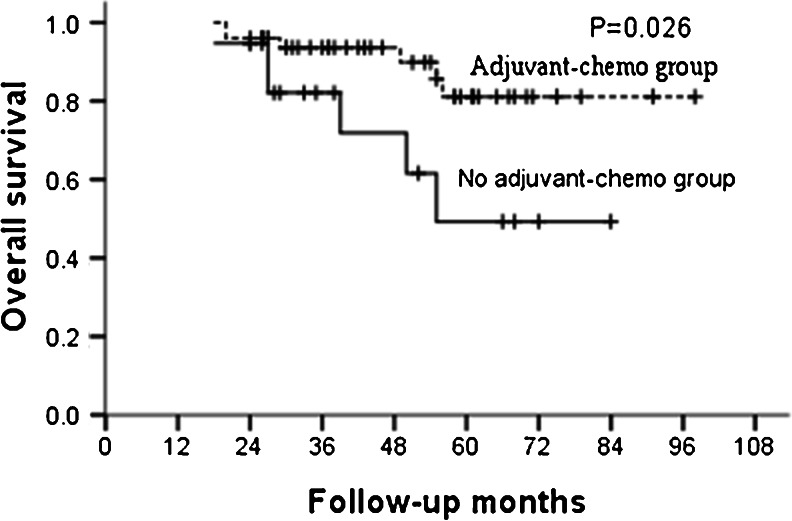
OS for the ypT3–4N0 patients stratified by treatment with adjuvant chemotherapy. The OS was significantly higher in adjuvant-chemotherapy group than that in no adjuvant-chemotherapy group for ypT3–4N0 patients (*P* = 0.026)

**Fig. 6:**
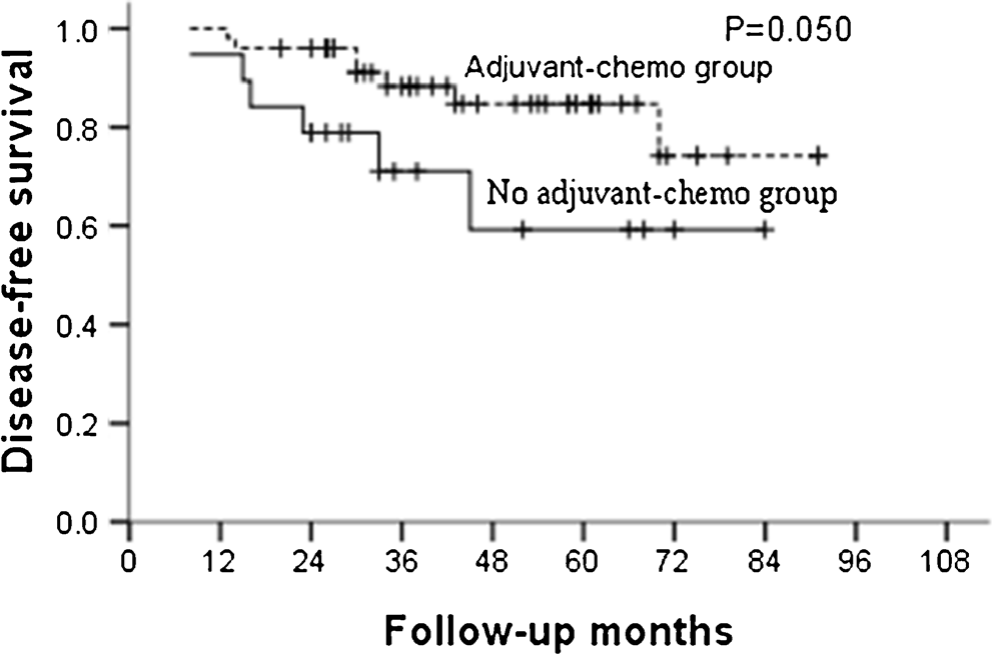
DFS for ypT3–4N0 patients stratified by treatment with adjuvant chemotherapy. The DFS was significantly higher in adjuvant-chemotherapy group than that in no adjuvant-chemotherapy group for ypT3–4N0 patients (*P* = 0.050)

For the whole group, the median follow-up was 46 months and the 5-year OS and DFS in the adjuvant and non-adjuvant chemotherapy groups were 87.0, 85.5 % and 70.6, 67.4 %, respectively (Table 2; Figs. 1 and 2). Further analysis showed that the 5-year OS and DFS were similar between patients who did and did not receive adjuvant chemotherapy in the ypT0–2N0 subgroup (Table 3; Figs. 3 and 4). However, for patients with ypT3–4N0, receiving adjuvant chemotherapy had the tendency to acquire higher OS and DFS than those who did not (Table 4; Figs. 5 and 6).

**Table 2:** OS and DFS for patients with ypT0–4N0

Group	Adjuvant-chemo group		No adjuvant-chemo group		*P* value
	3-year	5-year	3-year	5-year	
OS	93.6 %	87.0 %	90.5 %	70.6 %	0.052
DFS	91.7 %	85.5 %	76.2 %	67.4 %	0.059

*Adjuvant-chemo* adjuvant chemotherapy, *DFS* disease-free survival, *OS* overall survival

**Table 3:** OS and DFS for patients with ypT0–2N0

Group	Adjuvant-chemo group		No adjuvant-chemo group		*P* value
	3-year	5-year	3-year	5-year	
OS	93.7 %	93.7 %	96.2 %	83.7 %	0.401
DFS	92.2 %	85.8 %	80.4 %	73.1 %	0.359

*Adjuvant-chemo* adjuvant chemotherapy, *DFS* disease-free survival, *OS* overall survival

**Table 4:** OS and DFS for patients with ypT3–4N0

Group	Adjuvant-chemo group		No adjuvant-chemo group		*P* value
	3-year	5-year	3-year	5-year	
OS	93.6 %	81.1 %	82.1 %	49.3 %	0.026
DFS	88.3 %	84.8 %	71.1 %	59.2 %	0.050

*Adjuvant-chemo* adjuvant chemotherapy, *DFS* disease-free survival, *OS* overall survival

### Recurrence analysis

**Fig. 7:**
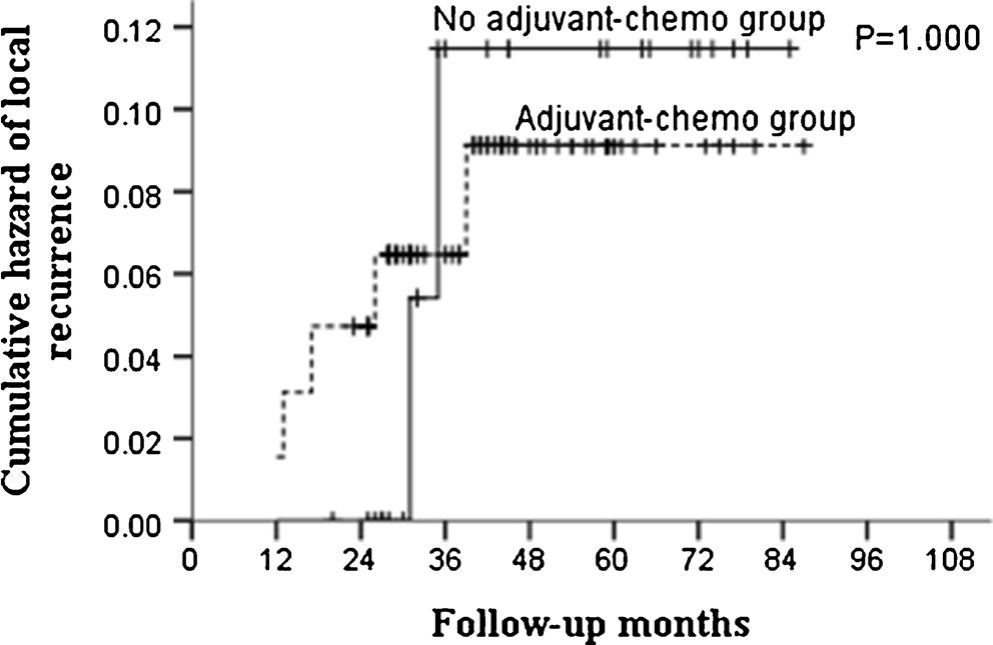
Cumulative hazard of local recurrence for ypT0–2N0 patients stratified by treatment with adjuvant chemotherapy. No significant difference was found in cumulative hazard of local recurrence between adjuvant-chemotherapy and no adjuvant-chemotherapy groups for ypT0–2N0 patients (*P* = 1.000)

**Fig. 8:**
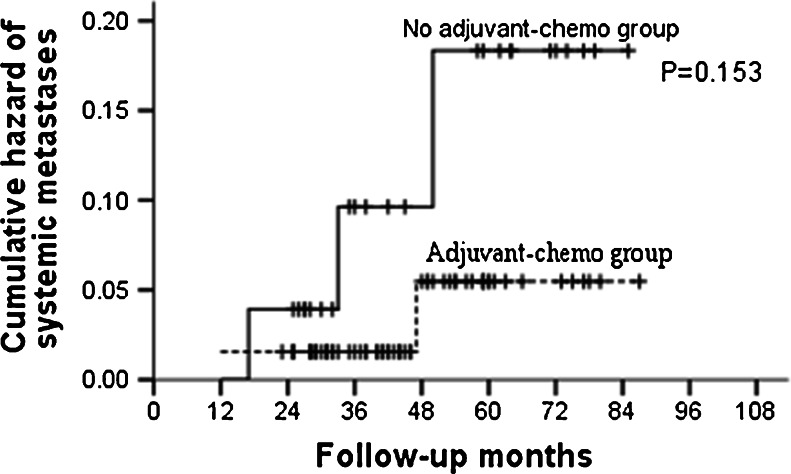
Cumulative hazard of systemic metastases for ypT0–2N0 patients stratified by treatment with adjuvant chemotherapy. No significant difference was found in cumulative hazard of systemic metastases between adjuvant-chemotherapy and no adjuvant-chemotherapy groups for ypT0–2N0 patients (*P* = 0.153)

**Fig. 9:**
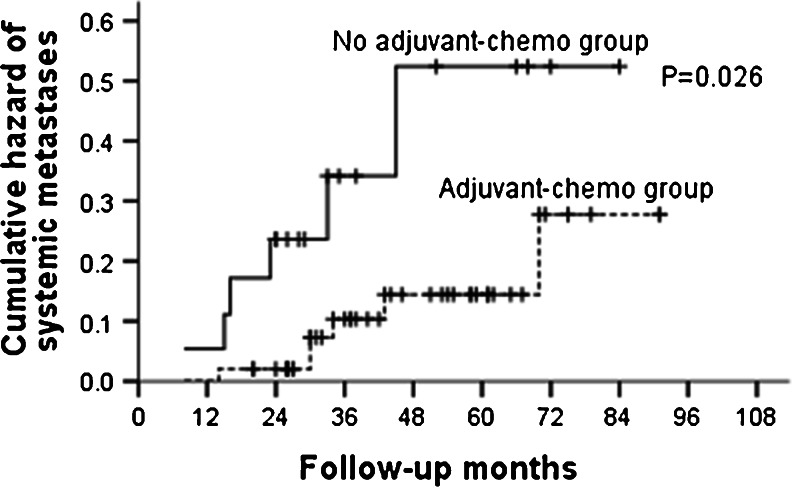
Cumulative hazard of systemic metastases for ypT3–4N0 patients stratified by treatment with adjuvant chemotherapy. The cumulative hazard of systemic metastases was significantly higher in no adjuvant-chemotherapy group than that in adjuvant-chemotherapy group for ypT3–4N0 patients (*P* = 0.026)

During the follow-up, 25 patients recurred. Among them, local failure occurred in eight patients, and distant failure occurred in 17 patients. For the subgroup of ypT0–2N0, no differences were found in both local and distant recurrences between patients who received adjuvant chemotherapy and those who did not (Table 5; Figs. 7 and 8). In ypT3–4N0 subgroup, though patients given no adjuvant chemotherapy did not show increased risk of local recurrence than those who were given, distant metastases rates were significant higher in these patients (Table 6; Fig. 9).

**Table 5:** Recurrence patterns for patients with ypT0–2N0

Group	Adjuvant-chemo group (*n* = 65)		No adjuvant-chemo group (*n* = 26)		*P* value
	3-year	5-year	3-year	5-year	
LR	4 (6.2 %)	5 (7.7 %)	2 (7.7 %)	2 (7.7 %)	1.000
SM	1 (1.5 %)	2 (3.1 %)	2 (7.7 %)	3 (11.5 %)	0.153

*Adjuvant-chemo* adjuvant chemotherapy, *LR* local recurrence, *SM* systemic metastases

**Table 6:** Recurrence patterns for patients with ypT3–4N0

Group	Adjuvant-chemo group (*n* = 50)		No adjuvant-chemo group (*n* = 19)		*P* value
	3-year	5-year	3-year	5-year	
LR	1 (2.0 %)	1 (2.0 %)	0 (0.0 %)	0 (0.0 %)	0.538
SM	4 (8.0 %)	6 (12.0 %)	5 (26.3 %)	6 (31.6 %)	0.026

*Adjuvant-chemo* adjuvant chemotherapy, *LR* local recurrence, *SM* systemic metastases

### Clinical predictors for DFS, LRFS, and DMFS

In multivariable analysis, both the final pathological stage and adjuvant chemotherapy were independent predictors of DMFS for the whole group (ypT0–4N0) but with no clinical factors found to be associated with LRFS (Table 7). When stratified by pathological stage, no clinical factor were found to predict that LRFS adjuvant chemotherapy was still significantly associated with DMFS with the adjusted HR of 0.297 (95 % CI 0.095 to 0.924, *P* = 0.036), in the ypT3–4 stratum (Table 8).

**Table 7:** Multivariate analyses of DFS, LRFS, and DMFS for ypT0–4 patients

Variable	DFS		LRFS		DMFS	
	HR (95 % CI)	*P* value	HR (95 % CI)	*P* value	HR (95 % CI)	*P* value
yp T stage						
ypT0–2 vs ypT3–4	NA		5.264 (0.648–42.791)	0.120	0.267 (0.094–0.761)	0.014
Adjuvant-chemo						
yes vs no	0.476 (0.216–1.050)	0.066	NA		0.294 (0.113–0.766)	0.012

*DFS* disease-free survival, *LRFS* local recurrence-free survival, *DMFS* distant metastasis-free survival, *NA* not available, *CI* confidence interval, *HR* hazard ratio, *Adjuvant-chemo* Adjuvant chemotherapy

**Table 8:** Multivariable analyses of DFS, LRFS, and DMFS for ypT3–4 patients

Variable	DFS		LRFS		DMFS	
	HR (95 % CI)	*P* value	HR (95 % CI)	*P* value	HR (95 % CI)	*P* value
yp T stage						
ypT0–2 vs ypT3–4	NA		NA		NA	
Adjuvant-chemo						
yes vs no	0.354 (0.118–1.057)	0.063	NA		0.297 (0.095–0.924)	0.036

*DFS* disease-free survival, *LRFS* local recurrence-free survival, *DMFS* distant metastasis-free survival, *NA* not available, *CI* confidence interval, *HR* hazard ratio. *Adjuvant-chemo* adjuvant chemotherapy

### Toxicity of chemoradiation and adjuvant chemotherapy

During the neoadjuvant chemoradiation, the most common toxicity types observed were diarrhea, neutropenia, and abdominal pain, and major severe adverse events (grade ≥ 3) included diarrhea (15.0 %), enteritis (12.6 %), and neurotoxity (5.6 %) (Table 9). However, the most common toxicity types for adjuvant chemotherapy were diarrhea, hand–foot syndrome, and neutropenia. Severe adverse events, during the adjuvant chemotherapy, were observed most in the toxicity types of diarrhea (17.4 %), nausea (4.3 %), and neurotoxity (4.3 %) (Table 10). No patents died of severe adverse events.

**Table 9:** Toxicity of chemoradiation

Toxicity type	Grade				
	1∼2	3	4	5	≥3
Abdominal distension	20 (12.5 %)	4 (2.5 %)	NA	NA	4 (2.5 %)
Abdominal pain	57 (35.6 %)	3 (1.9 %)	0	0	3 (1.9 %)
Nausea	40 (25 %)	6 (3.8 %)	0	0	6 (3.8 %)
Vomiting	33 (20.6 %)	5 (3.1 %)	0	0	5 (3.1 %)
Enteritis	30 (18.8 %)	18 (11.3 %)	2 (1.3 %)	0	20 (12.6 %)
Diarrhea	60 (37.5 %)	20 (12.5 %)	4 (2.5 %)	0	24 (15.0 %)
Constipation	44 (27.5 %)	5 (3.1 %)	3 (1.9 %)	0	8 (5.0 %)
Neurotoxity	34 (21.3 %)	9 (5.6 %)	0	0	9 (5.6 %)
Hand–foot syndrome	56 (35.0 %)	3 (1.9 %)	NA	NA	3 (1.9 %)
Hemoglobin	27 (16.9 %)	1 (0.6 %)	0	0	1 (0.6 %)
Neutrophils	73 (45.6 %)	7 (4.4 %)	0	0	7 (4.4 %)
Platelets	20 (12.5 %)	2 (1.3 %)	0	0	2 (1.3 %)

*NA* not available

**Table 10:** Toxicity of adjuvant chemotherapy

Toxicity type	Grade				
	1∼2	3	4	5	≥3
Abdominal distension	7 (6.1 %)	0	NA	NA	0
Abdominal pain	11 (9.6 %)	1 (0.9 %)	0	0	1 (0.9 %)
Nausea	30 (26.1 %)	5 (4.3 %)	0	0	5 (4.3 %)
Vomiting	20 (17.4 %)	3 (2.6 %)	0	0	3 (2.6 %)
Enteritis	7 (6.1 %)	2 (1.7 %)	0	0	2 (1.7 %)
Diarrhea	45 (39.1 %)	18 (15.7 %)	2 (1.7 %)	0	20 (17.4 %)
Constipation	28 (24.3 %)	1 (0.9 %)	0	0	1 (0.9 %)
Neurotoxity	18 (15.7 %)	5 (4.3 %)	0	0	5 (4.3 %)
Hand–foot syndrome	48 (41.7 %)	3 (2.6 %)	NA	NA	3 (2.6 %)
Hemoglobin	13 (11.3 %)	0	0	0	0
Neutrophils	46 (40.0 %)	2 (1.7 %)	0	0	2 (1.7 %)
Platelets	6 (5.2 %)	1 (0.9 %)	0	0	1 (0.9 %)

*NA* not available

## Discussion

In our present study, for patients with ypT0–4N0, 5-year OS and DFS in the adjuvant and no adjuvant-chemotherapy groups were 87.0, 85.5 % and 70.6, 67.4 %, respectively, with both the *P* values close to 0.050. Further subgroup analysis showed that postoperative adjuvant chemotherapy did not improve the survival for patients with ypT0–2N0, but for ypT3–4N0 patients, the 5-year OS were higher in the adjuvant-chemotherapy group than no in the adjuvant-chemotherapy group with also a tendency for 5-year DFS; meaning that the postoperative adjuvant chemotherapy may be clinically beneficial for patients with ypT3–4N0.

We then explored the patterns of recurrence for the entire group (ypT0–4N0) and subgroups (ypT0–2N0, ypT3–4N0). The results indicated similar local recurrence rates between patients who were given postoperative adjuvant chemotherapy and those who were not. However, the rates of distant metastases were found higher in no adjuvant-chemotherapy group compared to those in adjuvant-chemotherapy group for ypT3–4N0 patients. This was not shown in ypT0–2N0 patients; revealing that the real benefit of postoperative adjuvant chemotherapy lay in reducing the risk of distant metastasis for those with ypT3–4N0. To adjust other potentially measured confounders such as age and concurrent chemotherapy, multivariate analysis was performed by Cox proportional hazards regression. The results showed that both the final pathological stage and adjuvant chemotherapy were independent predictors of DMFS for the whole group (ypT0–4N0) and with no clinical factors associated with LRFS. After stratified by pathological stage, adjuvant chemotherapy was still significantly associated with DMFS in the ypT3–4N0 stratum, with patients who did not receive adjuvant chemotherapy having a 3.4-fold increased risk of distant metastasis relative to those who did.

The contemporary management of locally advanced rectal cancer is long-course chemoradiation followed by radical resection. No matter what the final pathology is, a full course of adjuvant chemotherapy is recommended. However, there is no sufficient evidence supporting this strategy [11]. The only clinical randomized controlled studies exploring the significance of chemotherapy given preoperatively or postoperatively for rectal cancer patients receiving preoperative radiotherapy failed to demonstrate that the postoperative adjuvant chemotherapy could significantly improve the OS and DFS [4]. The study group took a retrospective analysis subsequently with their results revealing that postoperative adjuvant chemotherapy could improve the 5-year OS and DFS for ypT0–2 patients but not for those with ypT3–4. [13] Their exploratory analysis suggested that same prognostic factor may drive both tumor sensitivity for primary treatment and long-term clinical benefit from further adjuvant chemotherapy. Thus, patients with good response to chemoradiation or radiotherapy may benefit from the later adjuvant chemotherapy, but they may devote all their attention to treatment response, failing to consider the prognosis of patients in the management of adjuvant chemotherapy. Patients with a good response to chemoradiation (ypT0–2N0) have been reported to acquire a favorable outcome regardless of receiving adjuvant chemotherapy or not. The contribution of adjuvant chemotherapy, though seemly effective, did little in improving the survival for ypT0–2N0 patients [8–10, 12]. Patients with ypT3–4 or N+ may need adjuvant chemotherapy for their increased risks of recurrence. Besides, the subsequent study was based only on the pathological T staging in the subgroup analysis and ignoring the pathological N stage, which was considered as a significant prognostic factor for rectal cancer patients who received CRT [15–18].

The role of adjuvant chemotherapy for ypN0 patients was first questioned by Fietkau et al [19]. In his study, disease-free survival for patients without lymph node metastases (ypN0) was excellent, independent of whether they had received postoperative chemotherapy. Similar results were as reported by Kiran ea al. [12], whose study included 128 rectal cancer cases with ypT0–4N0. Among these, 58 cases received adjuvant chemotherapy and 70 did not. The rates of local recurrence (*P* = 1.00), DFS (*P* = 0.41), and OS (*P* = 0.52) were comparable between two groups. Therefore, the author put forward a strong challenge to routinely administer adjuvant chemotherapy for postoperative rectal cancer patients with ypN0. Further subgroup analysis of ypN0 was performed by Huh JW et al. [9], who found that adjuvant chemotherapy did not significantly improve survival for ypT0–2N0 patients. They did not present the results for those with ypT3–4N0. The recurrence rates for ypN0 patients were analyzed by Govindarajan et al. [8], with the results of ypT0N0 2.7 %, ypT1–2N0 12.3 %, and ypT3–4N0 24.2 %. Though the 5-year DFS were comparable between patients receiving adjuvant chemotherapy and those who did not in subgroups of ypT0N0, ypT1–2N0, and ypT3–4N0, multivariable analysis showed pathological staging as the factor that was most strongly associated variable with recurrence (ypT3–4 vs ypT1–2, *P* < 0.0001) and that the value of adjuvant chemotherapy for ypT3–4N0 may need more investigation. Our present study showed that both the final pathological stage and adjuvant chemotherapy were significantly associated with distant metastasis for the whole group (ypT0–4N0) in the multivariable analysis. Thus, adjuvant chemotherapy, which was the only independent predictor of DMFS in ypT3–4 stratum when multivariable analysis of subgroups is performed, may be especially needed for the patients with final pathology of ypT3–4N0 to decrease the rates of distant failure.

NCCN recommends 4–6 months of postoperative adjuvant chemotherapy for all the locally rectal cancer patients receiving CRT and with the possible considerations that neo-CRT is a loco-regional therapy that should not, theoretically, control the potential of distant metastasis and that the routine administration of adjuvant chemotherapy for the patients in the seminal randomized trials, our results seemed to disfavor its use for patients with ypT0–2N0 due to its failing in improve survival significantly for these patients. If our conclusions were true, adjuvant chemotherapy may be avoided in approximately 47.0 % patients [6] who received CRT. This could significantly reduce the toxicity caused by chemotherapy [20–22]. Accordingly, we recommend selective administration of adjuvant chemotherapy for patients with high recurrence risks rather than those with fine prognosis needing further randomized trials to confirm since our conclusions were based on a small sample analysis.

Limitations such as small sample size, short follow-up, and retrospective design were not avoided in our study. Another major problem was the inconformity of regimens and courses of adjuvant chemotherapy, which prevented us from doing further analyses. We had difficulty in excluding the possibility that no better survival was found in ypT0–2 patients receiving adjuvant chemotherapy than those who did not. This was due to the inadequate courses of adjuvant chemotherapy, since the median month of adjuvant chemotherapy was four, but we noticed that seven cases in the ypT0–2N0 subgroup, who had developed recurrence in the adjuvant chemo-group, received at least 4 months of adjuvant chemotherapy, accompanied by five cases with recurrence in the no adjuvant-chemotherapy group. This may support our hypothesis that this was due to the adjuvant chemotherapy itself contributing little or no effect on the survival for ypT0–2N0 patients rather than the courses of it. It was also hard to decide whether the regimen of Xelox could result in a better outcome than that of Folfox6 for ypT3–4N0 patients, which may be analyzed by further subgroup analysis, but with too few cases in each group and unbalanced baseline characteristics between groups, it limited our ability to reveal the possible clinically significant differences.

In conclusion, according to the results of our study and other reports, for locally advanced rectal cancer after CRT and surgery, there is no sufficient evidence supporting that postoperative adjuvant chemotherapy could improve survival for ypT0–2N0 patients. However, adjuvant chemotherapy may be clinically meaningful for ypT3–4N0 patients by decreasing rates of distant metastases, thus leading to a better DFS and OS. We strongly suggest selective use of adjuvant chemotherapy for ypN0 patients. Further randomized controlled clinical trials are needed to address this problem.
